# Functionally Assessing the Age-Related Decline in the Detection Rate of Photons by Cone Photoreceptors

**DOI:** 10.3389/fnagi.2021.744444

**Published:** 2021-12-07

**Authors:** Asma Braham chaouche, Maryam Rezaei, Daphné Silvestre, Angelo Arleo, Rémy Allard

**Affiliations:** ^1^School of Optometry, Université de Montréal, Montréal, QC, Canada; ^2^Department of Educational and Counselling Psychology, McGill University, Montréal, QC, Canada; ^3^INSERM, CNRS, Institut de la Vision, Sorbonne Université, Paris, France

**Keywords:** photoreceptors, cones, photon noise, contrast sensitivity, motion, noise, equivalent input noise, detection of photons

## Abstract

Age-related decline in visual perception is usually attributed to optical factors of the eye and neural factors. However, the detection of light by cones converting light into neural signals is a crucial intermediate processing step of vision. Interestingly, a novel functional approach can evaluate many aspects of the visual system including the detection of photons by cones. This approach was used to investigate the underlying cause of age-related visual decline and found that the detection rate of cones was considerably affected with healthy aging. This functional test enabling to evaluate the detection of photons by cones could be particularly useful to screen for retinal pathologies affecting cones such as age-related macular degeneration. However, the paradigm used to functionally measure the detection of photons was complex as it was evaluating many other properties of the visual system. The aim of the current mini review is to clarify the underlying rationale of functionally evaluating the detection of photons by cones, describe a simpler approach to evaluate it, and review the impact of aging on the detection rate of cones.

## Introduction

Age-related macular degeneration (AMD) is a widespread age-related retinal pathology that can severely impair vision by progressively damaging the macula of the retina responsible for central vision. A study on AMD patients found a decline in the ability of retinal photoreceptors to detect light years before the atrophic changes occur (Elsner et al., 2002), which suggests that functional consequences of AMD may be detectable before structural changes (Elsner et al., 2002; Owsley, 2016). Thus, a decline in visual functions could be a useful predictor of vision loss. A functional test sensitive to a decline in the number of photons detected by cones could have a potential prognostic value to aid earlier diagnosis and intervention to slow down the progression of the vision loss.

We recently developed a psychophysical paradigm to evaluate many components of the visual system (Silvestre et al., 2018) including the modulation transfer function of the eye (MTF), the number of photons detected by cones, and different sources of neural noise. This paradigm was used to investigate the impact of healthy aging on various components of the visual system (Silvestre et al., 2019; Braham chaouche et al., 2020), and the results suggest that healthy aging has a considerable impact on the number of photons detected by cones despite standard visual functions like visual acuity and contrast sensitivity being little affected. These results suggest that the number of photons detected by cones could be affected at early stages of some retinal pathologies despite visual acuity and contrast sensitivity being little affected. Thus, this new psychophysical paradigm enabling to assess the number of photons detected by cones could be useful to screen for retinal pathologies.

However, the quantification of the number of photons detected by cones was embedded into a complex psychophysical paradigm developed to evaluate many components of the visual system, which makes the evaluation of only the number of photons detected by cones unnecessarily complex. The first purpose of this mini review is to summarize and clarify how the number of photons detected by cones can be assessed based on two functional measures of contrast sensitivity. The second section reviews the impact of healthy aging on the number of photons detected by cones that was observed using this functional approach.

## Functionally Measuring the Number of Photons Detected by Cones

The current section shows how the number of photons detected by cones can be evaluated by measuring contrast sensitivity under two specific visual conditions. The basic rationale is that the number of photons detected by cones varies in time and this variability [referred to as photon noise (Pelli, 1990; Silvestre et al., 2018)] directly depends on the mean number of photons detected. Under some conditions, this variability is limiting contrast sensitivity and its impact can be quantified using a noise paradigm.

### Relation Between the Expected Number of Photons Detected and Its Variability

There is a direct relation between the expected number of photons detected by cones and the variability in the number of photons detected. Given that each photon entering the eye has a probability of being detected by a cone, the number of photons detected is stochastic: even when the luminance intensity is constant, the number of photons detected varies with time. This variability is a source of internal noise, named photon noise (Pelli, 1990), that could affect some visual functions like contrast sensitivity. The probability distribution of the number of events follows a Poisson distribution (Hecht et al., 1942; Mueller, 1951), so the variance in the number of photons detected (*V*) is equal to the expected number of photons detected (*N*):


(1) V=N.

Thus, knowing the variability in the number of photons detected by cones indicates the number of photons detected on average.

### Impact of Photon Noise on Contrast Sensitivity

If the variation in the number of photons detected by cones (i.e., photon noise) were the only source of internal noise, then contrast sensitivity would be expected to be proportional to the square root of the luminance intensity (Silvestre et al., 2018), which corresponds to the de Vries-Rose law (de Vries, 1943; Rose, 1948). Indeed, since the standard deviation (σ) is equal to the square root of the variance


(2) $$\sigma =\sqrt{V},$$

then the standard deviation in the number of photons detected in a Poisson distribution is equal to the square root of the expected number of photons detected:


(3) $$\sigma =\sqrt{N}.$$

Given that the number of photons detected by cones is proportional to the retinal luminance intensity (*L*)


(4) N⁢∝⁢L,

then the standard deviation in the number of photons detected is proportional to the square root of the luminance intensity


(5) $$\sigma \,\text{∝}\,\sqrt{L}.$$

In contrast units (i.e., relative to the luminance intensity), the standard deviation would be inversely proportional to the square root of the luminance intensity


(6) $$\sigma_{c\,o\,n\,t\,r\,a\,s\,t}=\sigma /L,$$

and


(7) $$\sigma_{c\,o\,n\,t\,r\,a\,s\,t}\,\text{∝}\,1\,/\,\sqrt{L}.$$

Consequently, if the variability in the number of photons detected were the only source of internal noise affecting contrast sensitivity, contrast sensitivity would be proportional to the square root of the luminance intensity (Pelli, 1990; Raghavan, 1995; Silvestre et al., 2018).

### The Dominant Source of Noise

**FIGURE 1:**
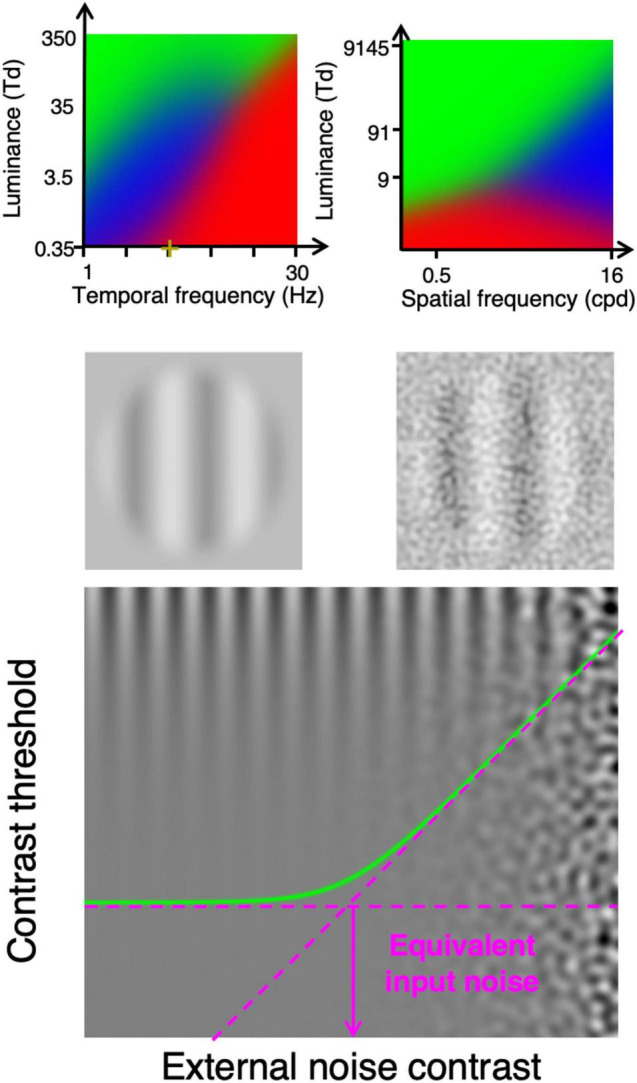
Top row: Dominant noise source identified based on psychophysically measuring contrast sensitivity as a function of luminance intensity. The blue area shows the viewing conditions (luminance intensity and temporal frequency for left graph (Allard and Arleo, 2017), luminance intensity and spatial frequency for right graph (Silvestre et al., 2018) at which the de Vries-Rose law was observed suggesting that the limiting internal noise source is photon noise (variability in the number of photons detected by cones). The green and red areas show conditions in which the Weber law and the linear law were observed, respectively. Second row, stimuli example used to functionally evaluate the detection of light by cones. Bottom graph shows contrast threshold as a function of external noise contrast, which can be used to evaluate the impact of the internal noise (equivalent input noise).

Under some visual conditions, the variability in the number of photons detected by cones (i.e., photon noise) is the dominant noise limiting contrast sensitivity. At high luminance intensities, contrast sensitivity is generally independent of luminance intensity (i.e., Weber law; Pelli, 1990; Silvestre et al., 2018), which suggests that it is limited by a source of noise that is independent of luminance intensity (e.g., late noise; Rose, 1948; Pelli, 1990). At low luminance intensities, contrast sensitivity is often proportional to luminance intensity (linear law; Silvestre et al., 2018), which suggests that contrast sensitivity is limited by another source of noise (e.g., early noise). Under some conditions, however, contrast sensitivity is proportional to the square root of the luminance intensity (i.e., de Vries-Rose law), which suggests that contrast sensitivity is limited by the variability in the number of photons detected by cones. For static stimuli, the de Vries-Rose law was observed at relatively high spatial frequencies (Figure 1, top right graph) (Silvestre et al., 2018). For dynamic stimuli at low spatial frequencies, the de Vries-Rose law was observed at relatively low temporal frequencies (Figure 1, top left graph) (Kelly, 1972). Consequently, under specific conditions, contrast sensitivity would be limited by the variability in the number of photons detected by cones (Pelli, 1990; Allard and Arleo, 2017; Silvestre et al., 2018).

### Quantifying the Impact of the Dominant Internal Noise

Interestingly, the impact of the internal noise can be functionally evaluated using a noise paradigm (Pelli, 1981, 1990; Pelli and Farell, 1999) consisting in measuring contrast sensitivity with different levels of visual noise added to the display (Figure 1, bottom graph). If the noise added to the display has a weaker impact on contrast sensitivity than the noise within the visual system, then its impact would be negligible (flat asymptote in Figure 1, bottom graph). Conversely, if the noise added to the display has a greater impact than the noise within the visual system then it would considerably affect contrast sensitivity (rising asymptote). The external noise level at which the noise starts to considerably affect contrast sensitivity (knee point of the curve in Figure 1, bottom graph), the external noise has the same impact on contrast sensitivity as the internal noise. Thus, the impact of the internal noise can be quantified as the level of external noise having the same impact, which is referred to as the equivalent input noise (Pelli, 1981, 1990).

Conveniently, measuring contrast threshold with and without noise (Figure 1, second row) is sufficient to derive the equivalent input noise (Pelli and Farell, 1999). Indeed, given that the log-log slope of the two asymptotes is 0 for the flat asymptote and 1 for the rising asymptote (contrast threshold proportional to the noise contrast, bottom graph of Figure 1), evaluating the two asymptotes is sufficient to derive the noise level at which they intersect, which corresponds to the equivalent input noise.

Measuring the equivalent input noise under conditions in which contrast sensitivity is limited by photon noise would quantify its impact on contrast sensitivity. In other words, it is possible to quantify the amount of external noise that has the same impact on contrast sensitivity as the variability in the number of photons detected by cones (i.e., photon noise).

However, the impact of an internal noise source on contrast sensitivity does not only depend on the level of the noise, it can also be modulated by a contrast gain preceding this noise source (Lu and Dosher, 2008; Silvestre et al., 2018). Indeed, a contrast gain preceding an internal noise source affects the signal contrast without affecting the noise contrast, which increases the relative impact of the noise.

Nonetheless, the detection of light by cones is not preceded by any neural processes, so the only contrast gain preceding the detection of photons by cones is due to optical factors: the MTF of the eye, which reduces the contrast of high spatial frequencies. Thus, if the signal is composed of only low spatial frequencies that are not noticeably affected by the MTF (i.e., retinal image at the same contrast as the stimulus), then the contrast of the signal would not be affected prior to the detection of photons by cones. For static stimuli, contrast sensitivity was found to be limited by the variability in the number of photons detected by cones at high spatial frequencies (top right graph in Figure 1). For moving stimuli composed of low spatial frequencies, contrast sensitivity can be limited by the variability in the number of photons detected by cones at low temporal frequencies and low luminance intensities (top left graph in Figure 1). Consequently, by measuring motion contrast sensitivity with and without external noise at a specific spatial frequency, temporal frequency and luminance intensity (e.g., 0.5 cycles per degree, 2 Hz and 10 Td) enables to quantify the variability in the number of photons detected by cones, which is equal to the number of photons detected by cones on average. Thus, the measure of the equivalent input noise under these conditions would directly and only depend on the number of photons detected by cones and should not depend on other processing factors; an observer with a lower detection rate would have a greater equivalent input noise under these viewing conditions.

## The Impact of Healthy Aging on the Detection Rate of Photons by Cones

We recently used the psychophysical paradigms developed to evaluate various components of the visual system (Allard and Arleo, 2017; Silvestre et al., 2018) to investigate the effect of healthy aging on various processing components of the visual system. Interestingly, the components that was the most affected with aging was the detection of photons by cones (i.e., photon noise).

**FIGURE 2:**
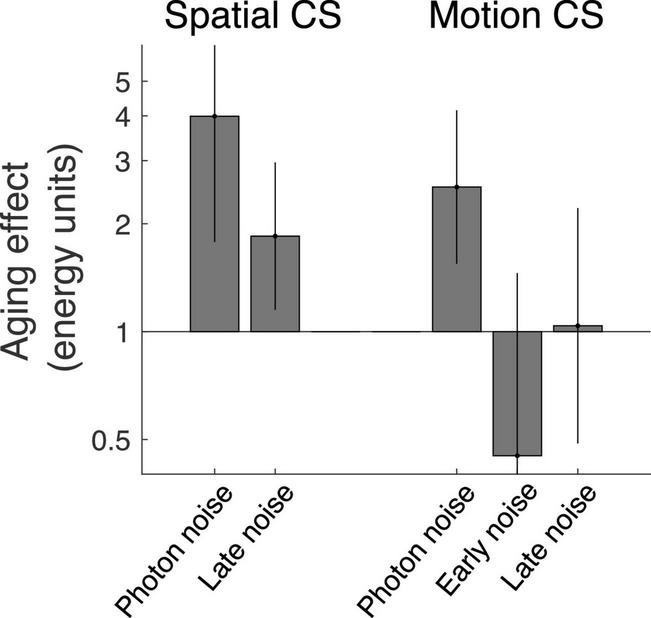
Impact of healthy aging on components of the visual system assessed using a novel noise paradigm for spatial contrast sensitivity (left; Silvestre et al., 2019) and motion contrast sensitivity (right; Braham chaouche et al., 2020). The photon noise represents the variability in the number of photons detected by cones and directly reflects the number of photons detected by cones (i.e., when contrast sensitivity is proportial to the square root of the luminance intensity (de Vries-Rose law). The early noise represents the impact of the dominant noise source for conditions under which contrast sensitivity is proportional to luminance intensity (linear law). The late noise represents the impact of the dominant noise source for conditions under which contrast sensitivity is independent of luminance intensity (Weber’s law).

In the first study (Silvestre et al., 2019), we investigated the impact healthy aging on spatial contrast sensitivity (i.e., static stimuli) at various spatial frequencies. This study suggested that from 26.5 to 75.9 years old, the detection rate of photons by cones dropped by a factor about 4 (i.e., photon noise increased by a factor of 4, Figure 2 left graph). In the second study (Braham chaouche et al., 2020), we evaluated the impact of healthy aging on motion contrast sensitivity at various temporal frequencies. This study suggested that from 26.5 to 75.7 years old, the detection rate of photons by cones dropped by a factor about 2.5 (i.e., photon noise increased by a factor of 2.5, Figure 2 right graph).

A reduction in the number of photons detected by cones does not necessarily imply that cones detected less photons as it could also be the result of less light reaching the retina due to optical factors like the pupil size or cataract development of the lens. However, in these two studies, the age-related declines in the detection rate of photons by cones were not due to age-related miosis (i.e., smaller pupil) because observers were wearing an artificial pupil (2.5 and 3 mm for the two studies, respectively) equating the number of photons entering the eye. Furthermore, the lower photon detection rate cannot be entirely due to cataract development of the lens reducing the number of short-wavelength photons reaching the retina, because a similar age-related effect was observed even when using only high wavelengths that are little affected by cataract development of the lens. Consequently, these two recent studies suggest that the age-related decline in the detection of photons by cones is not explained by a reduced retinal luminance intensity.

Another ocular factor to consider is intraocular scattering caused by cataract development of the lens, which affects the MTF of the eye by reducing the contrast of high spatial frequencies (Owsley, 2011; Martínez-Roda et al., 2016). In the first study, photon noise was the dominant noise only at high spatial frequencies, so the MTF of the eye had a direct impact on the equivalent input noise. However, the noise paradigm estimated the MTF of the eye, which was subtracted from the equivalent input noise to estimate the photon noise. Thus, the photon noise was theoretically not affected by intraocular scattering affecting high spatial frequencies. Nevertheless, any errors in the estimate of the age-related effect on the MTF would affect the calculated photon noise. In the second study, however, the signal was composed of only low spatial frequencies (0.5 cycles per degree) so the MTF of the eye was expected to have a negligible impact (Watson, 2013). Indeed, an age-related contrast gain reduction due to ocular factors is expected only at high spatial frequencies (Owsley, 2011; Martínez-Roda et al., 2016). Furthermore, any contrast loss due to optical factors would also affect the impact of neural noise, but no significant age-related effect on neural noise was observed (Figure 2, right). In other words, an age-effect on the equivalent input noise was observed to moderate luminance intensities (i.e., when photon noise dominated) and not to high luminance intensities (i.e., when late neural noise dominated). Thus, measuring photon noise at low spatial frequencies using motion contrast sensitivity makes the estimate of the photon noise more direct and should be more accurate. To avoid worrying about the MTF of the lens, photon noise should be measured at low spatial frequencies, which is possible using motion contrast sensitivity.

## Discussion

The current mini review highlights a new approach to non-invasively evaluate the detection rate of photons by cones. This functional approach enables to address a crucial visual process: the transduction of light into neural signals by cones. By measuring contrast sensitivity with and without external noise in specific conditions in which the variability in the number of photons detected by cones is limiting contrast sensitivity, the number of photons detected by cones can be evaluated. The current review therefore provides a simple functional paradigm to evaluate the efficiency of cones to detect light.

Other traditional techniques are also sensitive to the number of photons detected. For instance, imaging densitometry (e.g., Rushton and Henry, 1968) and subjective color-matching techniques (e.g., Burns et al., 1987) are sensitive to cone photopigments required in the absorption of photons. Such techniques were used to investigate the impact of aging on cone photopigments and the results were consistent with an age-related decline in the number of photons detected by cones (Keunen et al., 1987; Swanson and Fish, 1996). An advantage of the noise paradigm is that measuring the photon noise only requires measuring contrast sensitivity under two specific conditions, which only requires a few minutes without requiring a long light adaptation phase. Further studies are required to compare these techniques directly.

When studying age-related physiological alterations in the retina, it is often relevant to seek for functional correlates to quantify the impact of physiological alterations on visual functions. Typically, visual functions (e.g., visual acuity or contrast sensitivity) are evaluated at high luminance intensities at which performance depends little on luminance intensity. For instance, contrast sensitivity saturates at high luminance intensities (Weber law), which implies that it is independent of the amount of light detected by cones. Consequently, a lower detection rate of light would likely have little impact on contrast sensitivity at high luminance intensities. The fact that all visual functions require the phototransduction of light into neural signals does not imply that a lower detection rate of light would necessarily affect performance. To address the ability of cones to detect photons, a visual function should be evaluated under conditions in which performance critically depends on the number of photons detected by cones. The current mini review shows that it is possible to evaluate the detection of light by cones by measuring contrast sensitivity under two specific visual conditions. This novel approach provides a new way to assess the efficiency of cones to detect light.

Surprisingly, healthy aging was found to have a considerable impact on the detection rate by cones. Some age-related physiological changes at the retinal level could explain the decline in detection rate of photons by cones. One possible age-related alteration is a morphological one: the degeneration of the rods with aging induces a loss of alignment of the cones which lose their support (Curcio et al., 1993; Panda-Jonas et al., 1995) and reduces their detection rate (Cunea and Jeffery, 2007). Another possible change occurring with aging is a molecular one: a high concentration of cGMP (a secondary messenger in phototransduction) inhibits phototransduction and thus cone activation (Angueyra and Rieke, 2013). Further studies are needed to investigate the physiological cause of the age-related decline in the detection of light by cones.

A common symptom in early AMD is the difficulty under dim light conditions, which is consistent with the hypothesis that early AMD affects the detection rate of photons (Elsner et al., 2002) despite standard functions evaluated under clinical conditions are not considerably affected (Owsley, 2016). Consequently, evaluating the detection rate of photons by cones using the functional approach described in the current review could be useful to detect earlier signs of AMD. By enabling an earlier diagnosis, this new tool could prevent the progression of the disease by providing the opportunity to implement therapies at earlier stages of the disease. Further studies are required to investigate if measuring the detection rate of light by cones could be useful to detect early signs of AMD.

## Author Contributions

AB, MR, and RA co-wrote the mini review. DS and AA proofread the review. All authors contributed to the article and approved the submitted version.

## Conflict of Interest

The authors declare that the research was conducted in the absence of any commercial or financial relationships that could be construed as a potential conflict of interest.

## Publisher’s Note

All claims expressed in this article are solely those of the authors and do not necessarily represent those of their affiliated organizations, or those of the publisher, the editors and the reviewers. Any product that may be evaluated in this article, or claim that may be made by its manufacturer, is not guaranteed or endorsed by the publisher.
